# The evidence-informed decision-making process of the National Essential Medicines List Committee and Essential Drugs Programme

**DOI:** 10.4102/jcmsa.v4i1.369

**Published:** 2026-03-27

**Authors:** Amanda Brewer, Janine Jugathpal, Kim MacQuilkan, Marc Blockman, Andrew Gray, Tamara Kredo, Reneé de Waal, Lesley Robertson

**Affiliations:** 1 Essential Drugs Programme, South African National Department of Health, Pretoria, South Africa; 2 Department of Medicine, Division of Clinical Pharmacology, University of Cape Town, Cape Town, South Africa; 3 Division of Pharmacology, Discipline of Pharmaceutical Sciences, University of KwaZulu-Natal, Durban, South Africa; 4 Health Systems Research Unit, South African Medical Research Council, Cape Town, South Africa; 5 Centre for Integrated Data and Epidemiological Research, School of Public Health, University of Cape Town, Cape Town, South Africa; 6 Department of Psychiatry, School of Clinical Medicine, University of the Witwatersrand, Johannesburg, South Africa

## Foreword

A scientifically grounded approach to medicine policy lies at the heart of an equitable, effective and sustainable health system. In South Africa, the development of the National Standard Treatment Guidelines (STGs) and the Essential Medicines List (EML) is anchored in a rigorous evidence-informed decision-making (EIDM) process that seeks to balance clinical effectiveness, safety, cost-effectiveness, feasibility and public health impact within the realities of the local context.

This article provides a timely and important overview of the EIDM framework that undergirds the work of the National Essential Medicines List Committee (NEMLC) and its technical subcommittees. By clearly outlining how evidence is identified, appraised, contextualised and translated into policy decisions, the authors offer readers valuable insight into a process that is often perceived as opaque but is foundational to rational medicine selection and use in South Africa.

Importantly, this contribution also signals the *Journal of the Colleges of Medicine of South Africa*’s (JCMSA) intention to introduce a series of invited articles under the category of Drug Reviews. These reviews are envisaged as authoritative, evidence-based pieces that aim to demystify the evaluation of medicines, while also illuminating other forms of medicine review relevant to the NEMLC and the national EML process. By doing so, they seek to enhance transparency, foster informed engagement and strengthen trust in regulatory and policy decision-making among clinicians, researchers, policymakers and the broader public.

As a journal committed to advancing clinical excellence, scholarly rigour and public interest medicine, JCMSA views this article as a foundational reference for future Drug Reviews. We anticipate that these invited contributions will not only deepen understanding of formulary decisions but also support evidence-based prescribing and rational medicine use across levels of care.

We commend the authors for this clear and accessible exposition of South Africa’s EIDM process and look forward to the dialogue and scholarship that this new article category will stimulate.

Soraya Seedat

Clint Hendrikse

## Purpose

This article aims to describe the process by which South Africa’s National STGs and EML are developed to inform, and to share key resources with, stakeholders and end-users. The process relies on the newly developed EIDM Manual for the STGs and EML,^1^ which follows the principles developed in the National Standards for EIDM to Develop Trustworthy Healthcare Guidance Products.^2^ The National Standards were developed by the National Department of Health (NDoH) together with the South African Medical Research Council and the University of Stellenbosch through the Evidence-to-Decision (E2D) collaboration, an initiative aimed at supporting and building capacity for healthcare decision-making through timely and responsive evidence production and translation for efficient, effective and equitable health services and systems.

## Introduction and context

The NEMLC is a non-statutory committee constituted in terms of the National Drug Policy (1996)^3^ and appointed by the Minister of Health. The NEMLC, supported by its Expert Review Committee (ERC), is mandated to select essential medicines for the public health sector through a structured, transparent and robust decision-making framework. The framework,^1,2^ which has evolved over time^4^ to strengthen transparency and trustworthiness, considers public health relevance, clinical need, safety, efficacy, effectiveness, affordability and implementation implications.

The NEMLC develops and continuously reviews the National STGs and EML, available on the NDoH website,^5^ which guide clinical practice and prescribing in all public sector health establishments and inform procurement decisions. The STGs and EML are foundational to healthcare delivery in South Africa’s public sector. They ensure that patients across the country receive evidence-based, cost-effective and equitable treatment, regardless of where they seek care. By standardising treatment nationally, the STGs and EML improve the quality of care and reduce irrational prescribing. While the EML covers all service levels in the public health sector, the STGs serve as its implementation mechanism at primary and secondary levels of care, providing clinical guidance to support accessible, quality health care and promote the rational use of essential medicines. Updates to the STGs and EML are supported by various technical assessments of the medicines, including evidence reviews and costing analyses. The NEMLC reports outline the rationale for changes to existing documents.

Periodic review of the STGs and EML is conducted in consultation with the relevant NDoH Clinical Programmes, provincial health departments and other relevant stakeholders. Although primarily directed at the public health sector, the STGs and EML are also relied upon when medical schemes’ reimbursement decisions are challenged. In that sense, they set a minimum standard of care.

It is important for clinicians to understand the structure of the NEMLC because it directly determines which medicines they are allowed to prescribe in the public sector and under what circumstances. Understanding the NEMLC ensures that clinicians prescribe rationally, legally, safely and cost-effectively, in line with national policy. This links directly to the importance of the STGs and EML, which guide everyday clinical decision-making.

## Structures and roles in the development of National Standard Treatment Guidelines and the Essential Medicines List

The Essential Drugs Programme (EDP) Oversight Group, as the secretariat, provides governance, administrative and technical support to the NEMLC and ERC. Its responsibilities include project planning, supporting meetings, ensuring transparent communication, overseeing topic prioritisation, evidence retrieval and synthesis, providing technical and policy support, maintaining the EML, monitoring medicine use, managing dissemination of information, mitigating stock-outs and assisting with resolving implementation queries. The Group undertakes technical work to support the committees including, but not limited to, medicine reviews and relevant costing analyses.

**FIGURE 1:**
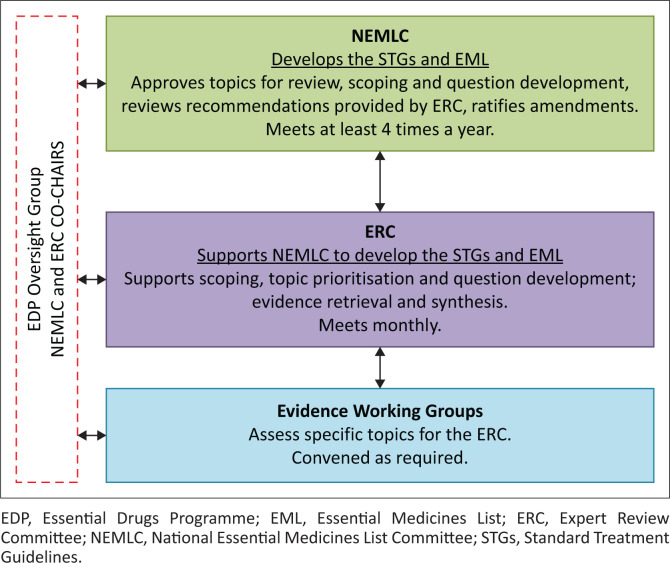
Structure of the National Essential Medicine List Committee and its Expert Review Committee.

The NEMLC serves as the final approval body for the STG and EML updates. Its functions include setting review priorities, considering ERC recommendations, approving amendments and ensuring decision governance quality. Membership comprises core experts (including ERC co-chairpersons, provincial representatives, as well as clinical and methodological experts across all levels of care) and ex-officio members from key health bodies and regulators. The ERC provides technical and scientific support to the NEMLC, including scoping, topic prioritisation, evidence retrieval and synthesis, and drafting STG and EML content. It comprises experts across all levels of care, subject matter and methods experts and co-opted members. Evidence Working Groups are established as required to lead evidence review and guidance updates for specific topics. The structure of the NEMLC and its ERC is provided in Figure 1.

## Governance, capacity-building and stakeholder engagement

The NEMLC and ERC operate in accordance with their Terms of Reference.^6^ All participants in NEMLC and ERC processes must adhere to the Affordable Medicines Directorate (AMD) Conflict of Interest Policy.^7^ Declarations are required at appointment, annually and before relevant meetings, for both financial and non-financial interests. All participants in the meetings and work of the committees must also comply with the NEMLC Confidentiality Guideline,^8^ ensuring integrity of the decision-making process.

Orientation of new members, ongoing skills training and capacity-building for NEMLC and ERC members are facilitated by the EDP Oversight Group with partner organisations. Capacity development is integral to institutionalising EIDM and ensuring the sustainability of the STGs and EML processes.

The EDP continuously maps and strives to engage diverse stakeholders in the STG and EML processes, including patient advocacy, healthcare providers, policymakers, programme managers, funders, manufacturers, regulators, academia and Health Technology Assessment (HTA) organisations. It is currently exploring ways to strengthen patient and public involvement and include people with lived experience in the decision-making process and guideline development. The EDP also promotes transparency and open communication by routinely publishing NEMLC-ratified documents on the NDoH NHI Webpage^9^ and disseminating updates through continuing professional development-accredited webinars, bulletins and other public-facing platforms.

## Evidence-informed decision-making process

**FIGURE 2:**
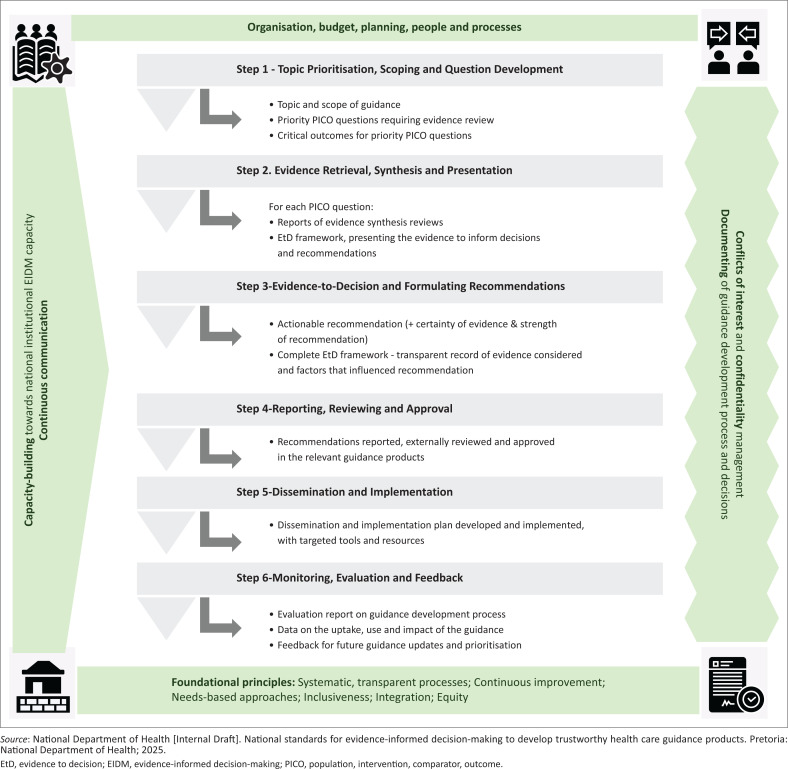
Standardised approach to support evidence-informed decision-making in the development of the Standard Treatment Guidelines and Essential Medicines List.

The EDP, NEMLC and ERC are guided by the methodology and processes outlined in the EIDM manual for the STGs and EML.^1^ Central to the process is the standardised approach, consisting of six core steps which were informed primarily by good practice methods and processes for HTA and guideline development aligned with global standards set out by the Grading of Recommendations Assessment, Development and Evaluation (GRADE) working group.^10^ These six core steps are provided in Figure 2.

## Conclusion

The NEMLC and EDP processes are not merely administrative mechanisms but form the backbone of South Africa’s evidence-informed medicine policy and a critical safeguard for public health. By anchoring decision-making in transparent governance, rigorous technical appraisal and meaningful stakeholder participation, the STGs and EML function as trustworthy national medicine guidance. Their continued strengthening is essential as they are central to ensuring equitable and rational access to essential medicines, defending limited health system resources from inefficiency and reinforcing the integrity of the South African public sector medicine selection process to enable universal health coverage and quality healthcare.
